# Genetic Variants of Coagulation Factor XI Show Association with Ischemic Stroke Up to 70 Years of Age

**DOI:** 10.1371/journal.pone.0075286

**Published:** 2013-09-25

**Authors:** Ellen Hanson, Staffan Nilsson, Katarina Jood, Bo Norrving, Gunnar Engström, Christian Blomstrand, Arne Lindgren, Olle Melander, Christina Jern

**Affiliations:** 1 Department of Clinical Neuroscience and Rehabilitation, Institute of Neuroscience and Physiology, the Sahlgrenska Academy at University of Gothenburg, Gothenburg, Sweden; 2 Department of Mathematical Statistics, Chalmers University of Technology, Gothenburg, Sweden; 3 Department of Clinical Sciences Lund, Neurology, Lund University, Lund, Sweden; 4 Department of Neurology, Skåne University Hospital, Lund, Sweden; 5 Department of Clinical Sciences Malmö, Lund University, Malmö, Sweden; University of Bonn, Institut of experimental hematology and transfusion medicine, Germany

## Abstract

**Background and Purpose:**

Coagulation factor XI (FXI) has an important role in the propagation and stabilization of a thrombus upon vessel injury. High FXI levels have been implicated in thrombotic diseases including ischemic stroke. The aim of our study was to investigate whether FXI gene (*F11*) variants are associated with ischemic stroke.

**Methods:**

The discovery sample, the Sahlgrenska Academy Study on Ischemic Stroke (SAHLSIS), included 844 patients with ischemic stroke and 668 controls, all aged 18-70 years. Replication was performed in the Lund Stroke Register (LSR) and Malmö Diet and Cancer study (MDC), together including 1213 patients and 788 controls up to 70 years of age, and in total 3145 patients and 1793 controls (18-102 years). Seven *F11* single-nucleotide polymorphisms (SNPs) were selected using a tagging approach.

**Results:**

The SNPs rs3733403, rs925451, and rs1593 showed independent associations with overall ischemic stroke in SAHLSIS, ORs of 0.74 (95% CI 0.59-0.94), 1.24 (95% CI 1.06-1.46), and 0.70 (95% CI 0.55-0.90), respectively. The association for rs925451 was replicated in the LSR and MDC sample in a pre-specified analysis of subjects aged 70 years or younger, OR of 1.16 (95% CI 1.00-1.34), whereas no SNP was replicated when all ages were included. In line with this, one *F11* haplotype was associated with overall ischemic stroke in the discovery sample and in the replication sample ≤70 years.

**Conclusions:**

We found significant associations between *F11* variation and overall ischemic stroke up to 70 years of age. These findings motivate further studies on the role of *F11* in ischemic stroke, especially in younger individuals.

## Introduction

Coagulation factor XI (FXI) was initially described as a member of the contact activation pathway which, in concert with the factor VII-tissue factor pathway, leads to thrombin generation, and subsequently the formation of a thrombus upon vessel injury. In recent years, studies have shown that thrombin activates FXI via a feedback loop [1]. FXI is now recognized as having an important role in sustained thrombin generation and formation of a stable thrombus [2].

FXI deficiency (Haemophilia C) is a rare bleeding disorder associated with a mild to moderate bleeding tendency [2]. In animal models, FXI deficiency confers protection from thrombus formation [3,4], and patients with this condition are protected against ischemic stroke (IS) and venous thrombosis (VT) [5,6], but not myocardial infarction [7]. Increased FXI levels have been suggested to be a risk factor for VT [8,9]. In studies of younger patients, elevated plasma levels or activity of FXI have been associated with an increased risk of IS, while plasma FXI does not appear to be of equal importance in myocardial infarction [10-14]. These studies provide support for the importance of FXI in thrombosis and, given the mild bleeding phenotype in FXI deficiency, FXI has gained recent attention as a potential target for anticoagulant therapy that could possibly reduce the risk of bleeding [15].

Interestingly, previous findings have pointed towards a relatively strong heritability for FXI plasma levels [16], and in a recent genome-wide genetic analysis of FXI activity levels, a significant association was detected for a variant in the FXI gene (*F11*) [17]. Moreover, single-nucleotide polymorphisms (SNPs) at the *F11* locus have been associated with both VT and FXI levels, and several of these *F11* SNPs were associated with VT independently of FXI levels [18,19]. Thus, *F11* variants might better reflect life-long FXI level exposure or they may even reflect other disease-associated mechanisms than those assessed with the plasma levels. There is no data on the possible influence of *F11* variants in IS. Therefore, we investigated whether *F11* variants are associated with IS and/or etiologic IS subtypes in the Sahlgrenska Academy Study on Ischemic Stroke (SAHLSIS), which comprises young and middle-aged IS patients and controls, i.e. 18-70 years of age. Replication was performed in a larger independent sample of patients with IS and controls. Because a previous study detected a relation between increased amounts of circulating active FXI and poor functional outcome after IS [20], we also investigated whether *F11* SNPs are associated with functional outcome after IS. The findings in the present study suggest that *F11* variants may be of importance in overall IS, possibly with a greater influence in younger individuals. 

## Subjects and Methods

### Study populations

The discovery sample comprised the participants in the case-control study SAHLSIS, which was designed to study genetic and hemostatic factors in IS. Sample characteristics, data collection and clinical definitions have been described [21,22]. In brief, white patients aged 18-70 years (n=844) who presented with IS were consecutively recruited at four stroke units in Western Sweden. White community controls aged 18-70 years (n=668), who were free from clinical atherothrombotic disease, were randomly selected to have a similar distribution with regard to age and sex as the cases. The upper age limit of 70 years was chosen based on studies showing that the influences of genetic and hemostatic factors are more pronounced in younger individuals [23,24]. The patients were classified into IS etiologic subtypes according to the TOAST criteria [25], using a local specified protocol as previously described [26]. Functional outcome at three months and at two years after index stroke was assessed according to the modified Rankin Scale (mRS) for the first 600 patients (for details, please see the Supplemental Methods in File S1).

The Lund Stroke Register (LSR) and the Malmö Diet and Cancer study (MDC) were used as a replication sample. Sample characteristics, data collection and clinical definitions have been described [27,28]. Briefly, LSR is a prospective, epidemiologic register which consecutively includes all patients with first-ever stroke from the local catchment area of Skåne University Hospital, Lund. For the current study, patients 18 years and older with first-ever IS between 2001 and 2009 were included. Control subjects were individuals without stroke, randomly selected from the same geographical uptake area, and age and sex matched to patients included during the first year (2001-2002) of the LSR project. MDC is a prospective, population based cohort study, which included 28449 randomly selected individuals at baseline examinations between 1991 and 1996. For the present study, incident cases of IS up to December 31th, 2006, were selected and matched (1:1) for age, sex and month of baseline examination in a nested case-control design. Control subjects were MDC participants who were alive and free from stroke at the time of the corresponding stroke event. At present, comparable TOAST subtype data as in SAHLSIS are not available for all patients in the LSR and MDC samples.

For a detailed description of the three study populations, please see the Supplemental Methods in File S1. All participants provided informed consent prior to enrolment. For participants who were unable to communicate, consent was obtained from their next-of-kin. For a vast majority of the participants, informed consent was obtained in writing from the participant or from a next-of-kin. If consent could not be obtained in writing, the verbal consent was carefully documented for each participant or next-of-kin. Reasons for obtaining verbal consent only included that the participant died before written consent could be obtained or was not able to fill in the written consent form and no relative was present who could assist. This study, including the procedure for obtaining consent, was approved by the IRB, i.e. the local ethics committee of Lund University. All studies were approved by the local ethics committees of the University of Gothenburg or Lund University.

### Genotyping

Genotype data from the CEU population in HapMap (release 23) was entered into the Haploview software 4.1, and seven tagSNPs were selected by TAGGER to capture the genetic variation within *F11* (chr4:187424112. 187447829, NCBI Genome Build 36) with minor allele frequency (MAF)>0.1 and r^2^>0.8. Genotyping was performed with the GoldenGate Assay (Illumina Inc., San Diego, CA, USA), and with KASP assays at KBioscience (Hertfordshire, UK). All genotyping was performed blinded to case/control status.

### Statistical analyses

Since SAHLSIS was designed to only include patients and controls up to 70 years of age, the statistical analyses performed in the replication sample primarily included subjects ≤70 years of age.

Hardy-Weinberg equilibrium (HWE) was assessed both in controls and cases. Associations between *F11* single SNPs or haplotypes and case/control status were investigated using an additive model in binary logistic regression, primarily adjusted for age, and sex (model A). A second logistic regression model also included hypertension, diabetes mellitus and smoking as covariates (model B). Age and sex were included as a proportion of the controls were matched with regard to these variables to the cases. Associations between *F11* SNPs and functional outcome were investigated by using an additive model in binary logistic regression, adjusted for the above covariates in addition to family history of stroke and TOAST subtype. Since the *F11* SNPs are in substantial linkage disequilibrium (LD), we used permutation tests with 10000 permutations to correct for multiple testing [29]. Corrected *P*-values are denoted *P*_c_. Assuming a multiplicative genetic model, the odds ratios (ORs) that can be detected with 80% power at the 5% level are in the range of 1.20-1.30, depending on the MAF (0.43-0.12) of the high-risk allele for overall IS in SAHLSIS. The corresponding ORs for the etiological subtypes are in the range of 1.49-1.68 for LVD, 1.40-1.58 for SVD, 1.42-1.60 for CE stroke, and 1.36-1.53 for cryptogenic stroke. Statistical softwares used were IBM SPSS Statistics version 20 for Windows (IBM Corporation, NY, USA) and HelixTree 6.3 (Golden Helix, Bozeman, MT, USA). The statistical significance level was 0.05 and *P*-values were two-tailed. 

## Results

Baseline characteristics for the discovery sample (SAHLSIS) and the replication sample (LSR and MDC) are shown in Table 1. As expected, the distribution of vascular risk factors was quite similar in the discovery sample and replication sample ≤70 years of age, whereas a smaller proportion of cases were males and smokers in the replication sample including subjects of all ages, compared to the discovery sample. As shown in Table 1, the median age is 64 years for cases in the replication sample ≤70 years compared to 59 years in SAHLSIS, which is due to that cases were 48-70 years in the MDC sample. In controls as well as in cases, the genotype distribution of each SNP did not deviate from that expected by HWE (*P*>0.05). The genotyping success rate was 98-100%.

**Table 1:** Baseline characteristics of the control and overall ischemic stroke groups in the discovery and replication samples.

	Discovery sample (SAHLSIS)		Replication sample (LSR, MDC ≤ 70 years)		Replication sample (LSR, MDC)	
	Control (n=668)	Ischemic stroke (n=844)	Control (n=788)	Ischemic stroke (n=1213)	Control (n=1793)	Ischemic stroke (n=3145)
Median age, years (IQR)	58 (50-64)	59 (51-64)	63 (59-67)	64 (60-68)	72 (65-79)	74 (66-81)
Male, n (%)	392 (59)	554 (66)	483 (61)	767 (63)	999 (56)	1661 (53)
Hypertension, n (%)	230 (34)	487 (58)	354 (45)	811 (67)	944 (53)	2143 (69)
Current smoking, n (%)	131 (20)	324 (38)	162 (21)	460 (38)	282 (16)	725 (24)
Diabetes, n (%)	33 (5)	153 (18)	30 (4)	222 (18)	93 (5)	641 (21)

SAHLSIS, the Sahlgrenska Academy Study on Ischemic Stroke; LSR, the Lund Stroke Register; MDC, the Malmö Diet and Cancer study. Data are shown as median and interquartile range (IQR), or number (n) and percentage (%)

### *F11* variation and overall IS

The observed genotype frequencies for the SNPs in the control and overall IS groups, as well as ORs and 95% confidence interval (CI) for overall IS are presented in Table 2 and in Table S1 in File S1. In the logistic regression model A, the minor alleles (G) of rs3733403 and (T) of rs1593 were significantly associated with a decreased risk for overall IS in the discovery sample, whereas the minor allele (A) of rs925451 was associated with an increased risk (Table 2). After inclusion of vascular risk factors in the regression model, all associations remained significant. After correction for multiple testing, the associations for rs1593 and rs925451 remained (*P*_c_<0.04).

**Table 2:** Genotype frequencies in the control and overall ischemic stroke groups in the discovery and replication samples, as well as ORs (95% CI) for overall ischemic stroke in these samples.

	Discovery sample (SAHLSIS)		Replication sample (LSR, MDC ≤ 70 years)		Replication sample (LSR, MDC)	
	Controls (n=668)	Ischemic stroke (n=844)	Controls (n=788)	Ischemic stroke (n=1213)	Controls (n=1793)	Ischemic stroke (n=3145)
rs3733403						
CC	500 (75)	669 (80)	576 (74)	918 (76)	1358 (76)	2409 (77)
CG	150 (23)	162 (19)	184 (24)	266 (22)	391 (22)	648 (21)
GG	15 (2)	8 (1)	17 (2)	18 (1)	27 (2)	55 (2)
OR (95% CI)‡	ref	0.77 (0.62-0.96)*	ref	0.88 (0.73-1.06)	ref	0.98 (0.86-1.11)
OR (95% CI)§	ref	0.74 (0.59-0.94)*	ref	0.86 (0.70-1.06)	ref	0.99 (0.86-1.12)
rs925451						
GG	243 (37)	263 (31)	308 (41)	459 (39)	673 (39)	1177 (38)
GA	327 (49)	418 (50)	353 (46)	542 (46)	805 (46)	1437 (47)
AA	94 (14)	158 (19)	99 (13)	189 (16)	259 (15)	469 (15)
OR (95% CI)‡	ref	1.23 (1.06-1.43)†	ref	1.11 (1.97-1.27)	ref	1.01 (0.93-1.10)
OR (95% CI)§	ref	1.24 (1.06-1.46)†	ref	1.16 (1.00-1.34)*	ref	1.02 (0.93-1.11)
rs1593						
AA	499 (75)	670 (80)	581 (76)	891 (75)	1313 (75)	2274 (74)
AT	155 (23)	165 (20)	170 (22)	279 (24)	408 (23)	762 (25)
TT	12 (2)	5 (1)	14 (2)	16 (1)	30 (2)	47 (2)
OR (95% CI)‡	ref	0.74 (0.59-0.92)†	ref	1.02 (0.84-1.24)	ref	1.04 (0.92-1.18)
OR (95% CI)§	ref	0.70 (0.55-0.90)†	ref	1.01 (0.82-1.25)	ref	1.03 (0.91-1.18)

SAHLSIS indicates the Sahlgrenska Academy Study on Ischemic Stroke; LSR, Lund Stroke Register; MDC, Malmö Diet and Cancer Study; OR, odds ratio; CI, confidence interval. An additive model in binary logistic regression was used. ‡ Adjusted for age, and sex. § Adjusted for age, sex, hypertension, diabetes mellitus, and smoking. *P<0.05, † *P*<0.01.

The seven *F11* SNPs are situated in one LD block (Figure S1 in File S1). The haplotype analysis was confined to rs3733403, rs925451 and rs1593, and we identified four haplotypes with a frequency >1% in the discovery sample (Table 3). Significant associations were observed for haplotype CAA with an increased risk of IS, and for haplotypes GGA, and CGT with a decreased risk, in regression model A. All associations remained in regression model B. Only the association with haplotype CAA was maintained after correction for multiple testing (*P*_c_<0.004). The haplotype analysis was congruent with the results for the single SNPs, for example, haplotype CAA comprises the major alleles (C) of rs3733403 and (A) of rs1593, and minor allele (A) of rs925451, and was associated with an increased risk of IS.

**Table 3:** Estimated haplotype frequencies and ORs with 95% CI for overall IS, as compared to controls, in the discovery sample and the replication sample.

	**Discovery sample**, SAHLSIS			**Replication sample**, LSR, MDC ≤ 70 years			**Replication sample**, LSR, MDC		
Haplotype	Estimated frequency (%)	OR (95% CI)‡	OR (95% CI)§	Estimated frequency (%)	OR (95% CI)‡	OR (95% CI)§	Estimated frequency (%)	OR (95% CI)‡	OR (95% CI)§
CAA	41.0	1.26 (1.08-1.46)†	1.27 (1.08-1.49)†	38.7	1.12 (0.98-1.28)	1.17 (1.02-1.36)*	37.3	1.02 (0.94-1.12)	1.03 (0.94-1.13)
CGA	35.3	1.03 (0.88-1.20)	1.05 (0.90-1.24)	-	-	-	-	-	-
GGA	11.9	0.77 (0.62-0.96)*	0.74 (0.58-0.94)*	12.6	0.89 (0.74-1.08)	0.89 (0.74-1.08)	12.3	0.98 (0.87-1.11)	1.00 (0.87-1.14)
CGT	11.2	0.75 (0.59-0.95)*	0.72 (0.55-0.92)*	11.8	1.02 (0.84-1.25)	1.01 (0.81-1.26)	13.0	1.06 (0.93-1.20)	1.05 (0.92-1.20)

IS, ischemic stroke; OR, odds ratio; CI, confidence interval. An additive model in binary logistic regression was used. ‡ Adjusted for age, and sex. § Adjusted for age, sex, hypertension, diabetes mellitus, and smoking. *P<0.05, †*P*<0.01

To investigate whether we could replicate the above findings in an independent sample of patients with IS up to 70 years of age, we genotyped rs3733403, rs1593, and rs925451 in LSR and MDC. The genotype frequencies are presented in Table 2. In this sample, an association was observed between rs925451 and overall IS in regression model B, whereas no association was detected for rs3733403 or rs1593 (Table 2). When participants of all ages were included, no association was observed for any of the three SNPs, either in the regression model A or when adjusting for vascular risk factors in model B (Table 2).

Estimated haplotype frequencies are displayed in Table 3. The haplotypes CAA, GGA and CGT that were associated with IS in SAHLSIS were investigated in the replication sample. In the logistic regression, haplotype CAA was associated with an increased risk of overall IS (Table 3). Similarly to the single SNP analysis, we found no association between any haplotype and IS in the LSR and MDC sample of all ages.

### *F11* variation and the etiologic subtypes of IS

The subtype analysis was confined to the etiologic subtypes large-vessel disease, small-vessel disease, cardioembolic stroke, and cryptogenic stroke. Genotype frequencies of the seven *F11* SNPs and ORs (95% CI) for the four major etiologic subtypes in SAHLSIS are displayed in Table S2 in File S1. For the three SNPs associated with overall IS in SAHLSIS, the ORs for all four subtypes showed a similar pattern as for the whole sample. This was also true for the other four SNPs. Hence, no subtype-specific differences were observed. The haplotype analysis did not add any further information.

### *F11* variation and functional outcome after IS

Of the first 600 patients in SAHLSIS, 130 patients were dependent or deceased (mRS score 3-6) and 438 patients had a favorable outcome (mRS score 0-2) at three months after index stroke, and the corresponding numbers were 134 and 458 patients at two years after index stroke (missing scores for 31 and 8 patients, respectively, at the two time-points). The minor allele (G) of rs4253423 was associated with a decreased risk of death or dependency, both at three months and at two years after index stroke (OR of 0.57, 95% CI 0.36-0.90, and OR of 0.56, 95% CI 0.36-0.88, respectively). None of the associations remained significant after correction for multiple testing (*P*_c_>0.08). No haplotype was associated with functional outcome at any of the two time-points. 

## Discussion

In the present study, we investigated variation at the *F11* locus and IS in the discovery sample SAHLSIS with replication in a larger independent sample including participants up to 70 years of age from the LSR and MDC studies. As far as we are aware, this is the first study examining *F11* variation in IS.

We found independent associations between three single SNPs (rs3733403, rs925451, and rs1593) and three haplotypes spanning these SNPs (CAA, CGA, and CGT) and overall IS in the discovery sample SAHLSIS. In the replication sample ≤70 years of age, weak associations were observed for rs925451 and the haplotype CAA, whereas no association was detected for the other variants. Of note is that the cases were 48-70 years in the MDC sample, which could potentially contribute to the weaker association observed in the replication sample compared to the discovery sample. The minor allele of rs925451 has previously been associated with an increased risk of VT [30]. This variant is located within the intronic region of *F11*, and is not known to be functional. Other studies have also reported associations between SNPs in *F11* and VT [18,19,30-32]. In these studies, the strongest associations were shown for the minor allele of rs2289252 and rs3756008, and the major allele of rs2036914, with an increased risk of VT. For rs2036914, we could not detect a significant association with overall IS. The SNPs rs2289252 and rs3756008 were not included in the present study, but rs2289252 is in strong LD with rs925451 (r^2^=0.87 in HapMap). We could not detect any associations between *F11* variants and overall IS in the LSR and MDC sample including participants of all ages. Altogether, these results suggest that variation within the *F11* locus might contribute to the pathophysiology of IS in younger rather than older subjects. Of relevance to the present results is that increased FXI plasma levels have been reported in young patients with IS compared to controls [10-12].

Regarding the etiologic subtypes of IS, we could not detect any subtype-specific associations for any of the *F11* variants. Due to the limited sample size of these subtypes in SAHLSIS and the fact that a similar subtype-specific analysis could not be performed in the replication sample, smaller effect sizes for the etiologic subtypes cannot be excluded. Moreover, we found no significant associations between *F11* SNPs and functional outcome (as measured by the mRS score) after overall IS. Thus, further studies on *F11* SNPs in the etiologic subtypes of IS and in relation to functional outcome after IS are clearly warranted.

The present study has the advantage of a homogenous sample with white participants from the southwest of Sweden. The sample size is relatively large, although, to detect small ORs an even larger sample size would be needed. Another limitation is that the plasma levels or activity of FXI were not examined, which restricts the speculation about possible mechanisms behind the detected associations. On the other hand, genetic variation can help identify other (perhaps more local) mechanisms in contrast to the systemic effects that are measured by plasma levels. In line with this, the previously reported association for rs2289252 (strong LD with rs925451) with VT was independent of FXI plasma levels [18], implying that other disease-associated mechanisms are also at play. Another explanation may be that rs2289252 (or a variant in LD) better reflects the life-long FXI level exposure than a single plasma measurement. A limitation of the present study is also that results regarding etiologic subtypes or functional outcome was not validated in an independent sample.

In conclusion, in this first study on FXI gene variants and overall IS we found weak but significant associations with IS up to 70 years of age, suggesting a role for FXI in IS especially at younger ages. However, these findings need to be further validated and future studies on FXI gene variation in IS are motivated. 

## Supporting Information

File S1 **Supporting files.**
Figure S1, Linkage disequilibrium (LD) plot of the seven tagSNPs in *F11*. One haplotype block was determined by the Solid Spine of LD algorithm. Graphic representation of the LD structure is based on pairwise D’. Table S1, Genotype frequencies, *n* (%), for four *F11* tagSNPs in the control and overall IS groups, as well as ORs and 95% CI for overall IS, as compared to controls. Table S2, Genotype frequencies, *n* (%), for the seven *F11* tagSNPs in controls and in the four major IS subtypes in the discovery sample SAHLSIS, as well as ORs and 95% CI for the subtypes.
(DOCX)
