# Supplementary material for: Genetic Variants of Coagulation Factor XI Show Association with Ischemic Stroke Up to 70 Years of Age
Source: PLoS One. 2013 Sep 25;8(9):e75286. doi: 10.1371/journal.pone.0075286 (PMC3783404; doi:10.1371/journal.pone.0075286)
Supplement: File S1 — Supporting files. Figure S1, Linkage disequilibrium (LD) plot of the seven tagSNPs in F11. One haplotype block was determined by the Solid Spine of LD algorithm. Graphic representation of the LD structure is based on pairwise D’. Table S1, Genotype frequencies, n (%), for four F11 tagSNPs in the control and overall IS groups, as well as ORs and 95% CI for overall IS, as compared to controls. Table S2, Genotype frequencies, n (%), for the seven F11 tagSNPs in controls and in the four major IS subtypes in the discovery sample SAHLSIS, as well as ORs and 95% CI for the subtypes. (DOCX) [file pone.0075286.s001.docx]

**SUPPORTING INFORMATION**

**Genetic variants of coagulation factor XI show association with ischemic stroke up to 70 years of age**

Ellen Hanson, Staffan Nilsson, Katarina Jood, Bo Norrving, Gunnar Engström, Christian Blomstrand, Arne Lindgren, Olle Melander, and Christina Jern.

**Supplemental Methods**

The manuscript was prepared according to the STROBE guidelines [[1](#_ENREF_1)].

**The Sahlgrenska Study of Ischemic Stroke (SAHLSIS)**

The study population comprised white patients (n=844) with first-ever (n=732) or recurrent (n=112) ischemic stroke (IS) who were consecutively recruited between 1998 and 2008 at four stroke units in Western Sweden. Healthy white population controls (n=668) were randomly selected to match cases with regards to age and sex. Controls were from the same geographical region as the patients, and they were recruited through a population-based health survey [[2](#_ENREF_2)] or from the Swedish Population Registry. IS was defined as an episode of focal neurological deficits with acute onset and lasting >24 hours or until death, with no apparent non-vascular cause, and no signs of primary hemorrhage on brain imaging. Information on the subjects’ vascular risk factors was collected as described elsewhere [[3](#_ENREF_3)]. Hypertension was defined by pharmacological treatment for hypertension, systolic blood pressure ≥ 160 mm Hg, and/or diastolic blood pressure ≥ 90mm Hg. Diabetes mellitus was defined by diet or pharmacological treatment, fasting plasma glucose ≥ 7.0 mmol/L, or fasting blood glucose ≥ 6.1 mmol/L. Smoking habit was coded as current versus never or former. Information about diabetes mellitus was missing in 2 participants, hypertension in 12, and smoking habits in 4.

All cases underwent ECG and neuroimaging with computed tomography (CT) and/or magnetic resonance imaging (MRI). Extracranial carotid and vertebral duplex ultrasound, MR angiography, catheter angiogram, transcranial Doppler ultrasound, transthoracic and/or transesophageal echo-cardiography were performed when clinically indicated. Based on clinical presentation and results from the diagnostic work-up, case were classified into IS etiologic subtypes according to modified Trial of Org 10172 in Acute Stroke Treatment (TOAST) criteria [[4](#_ENREF_4)]. In order to minimize interrater variability, the original TOAST criteria were refined according to a local protocol [[5](#_ENREF_5)]. Risk factors, other than atrial fibrillation and carotid stenosis (*i.e*. hypertension and diabetes), were not included in the protocol. Adjudication of subtypes was performed by two neurologists (KJ and CB). The distribution of subtypes was as follows; large-vessel disease (LVD, n=111), small-vessel disease (SVD, n=165), cardioembolic (CE) stroke (n=151), cryptogenic stroke (n=206), other determined cause of stroke (n=92), and undetermined stroke (n=119). Cryptogenic stroke was defined when no cause was identified despite an extensive evaluation. Undetermined stroke included cases for which more than one etiology was identified or when the evaluation was cursory.

Functional outcome at three months and at two years after index stroke was assessed according to the modified Rankin Scale (mRS) for the first 600 patients in SAHLSIS (missing scores for 31 and 8 patients, respectively, at the two time-points). At the 3-month follow-up, functional outcome was assessed through examinations by a physician trained in stroke medicine. At the 2-year follow-up, all surviving patients were contacted by a study nurse trained in stroke medicine for a structured telephone interview that, among other things, involved the assessment of functional outcome [[6](#_ENREF_6)]. As previously described [[3](#_ENREF_3)], the mRS score was dichotomized for death or dependency (mRS score 3-6) versus a favorable outcome (mRS 0-2).

All participants provided written informed consent prior to enrolment. For participants who were unable to communicate, consent was obtained from their next-of-kin. This study was approved by the Ethics Committee of the University of Gothenburg.

**The Lund Stroke Register (LSR) and the Malmö Diet and Cancer study (MDC)**

Sample characteristics, data collection and clinical definitions for LSR and MDC have been described [[7](#_ENREF_7),[8](#_ENREF_8)]. IS was defined as in SAHLSIS, and all patients underwent neuroimaging or autopsy. Hypertension was defined by pharmacological treatment for hypertension, systolic blood pressure ≥160 mmHg, and/or diastolic blood pressure ≥ 90 mmHg. Diabetes mellitus was defined by diet or pharmacological treatment, fasting plasma glucose ≥7.0 mmol/L, and/or fasting blood glucose ≥6.1 mmol/L, or self-reported diabetes. Smoking history was coded as current versus never or former. In the combined sample of LSR and MDC ≤70 years of age, 28 participants had missing data for diabetes mellitus, 17 for hypertension, and 34 for smoking habit. The corresponding numbers for the whole sample including participants of all ages were 111 for diabetes mellitus, 77 for hypertension, and 87 for smoking habit.

LSR is a prospective, consecutive hospital based case-control study that has been described previously [[7](#_ENREF_7)]. All patients with first-ever stroke from the local uptake area of Skåne University Hospital, Lund receiving hospital attention are included since 2001 in this study. For the current study, patients 18 years and older with first-ever ischemic stroke between 2001 and 2009 were included if they (or when they were not being able to communicate, their next-of-kin) gave informed consent to participate and blood samples for DNA collection were taken. All patients underwent CT, MR or post-mortem examination of the brain. Controls subjects were age and gender matched to patients recruited during the first year of LSR (2001-2002), and were from the same geographical uptake area.

MDC is a prospective, population based cohort study, which was established with the aim of studying the relationships between diet and health outcomes [[8](#_ENREF_8),[9](#_ENREF_9)]. A total of 28449 individuals, 45-73 years of age, participated in the baseline examinations between 1991 and 1996. Incidence of stroke after the baseline examination has been followed by data linkage with national and local registers. Validation of the stroke diagnosis by review of hospital records has been performed for 95% of all IS in the cohort. The procedures for retrieval of incident cases of stroke, case ascertainment and stroke classification have been reported elsewhere [[9](#_ENREF_9)]. For the purpose of studying genetic determinants of IS in MDC, incident cases of IS up to December 31th, 2006, were selected and matched (1:1) for age, sex and month of baseline examination in a nested case-control design. Control subjects were MDC participants who were alive and free from stroke at the time of the corresponding stroke event. Risk factors were assessed at the examination 1991-1996.

All participants provided informed consent prior to enrolment. For participants who were unable to communicate, consent was obtained from their next-of-kin. The studies were approved by the Ethics Committee of Lund University.

**Supplemental Results**

**Genetic variation in *F11* not investigated in the replication sample**

Genotype frequencies for the overall IS and control groups in SAHLSIS for the four *F11* tag single-nucleotide polymorphisms (tagSNPs) that were not investigated in the replication sample, are presented in Table S1. In the same Table, odds ratios (ORs) and 95 % confidence intervals (CI) for overall IS, as compared to controls, are shown.

| **Table S1.** Genotype frequencies, *n* (%), for four *F11* tagSNPs in the control and overall IS groups, as well as ORs and 95% CI for overall IS, as compared to controls. | | | |
| --- | --- | --- | --- |
|  |  | **Control**  (n=668) | **Ischemic stroke**  (n=844) |
| rs2036914 | GG | 204 (31) | 288 (34) |
|  | GA | 332 (50) | 398 (47) |
|  | AA | 130 (20) | 153 (18) |
|  | OR (95% CI)* | ref | 0.9 (0.8-1.0) |
|  | OR (95% CI)† | ref | 0.9 (0.8-1.0) |
| rs4253423 | AA | 474 (71) | 596 (72) |
|  | AG | 173 (26) | 216 (26) |
|  | GG | 18 (3) | 20 (2) |
|  | OR (95% CI)* | ref | 1.0 (0.8-1.2) |
|  | OR (95% CI)† | ref | 1.0 (0.8-1.2) |
| rs3822058 | GG | 302 (45) | 391 (47) |
|  | GA | 293 (44) | 368 (44) |
|  | AA | 71 (11) | 80 (10) |
|  | OR (95% CI)* | ref | 0.9 (0.8-1.1) |
|  | OR (95% CI)† | ref | 0.9 (0.8-1.1) |
| rs4253431 | GG | 506 (76) | 643 (77) |
|  | GA | 150 (23) | 183 (22) |
|  | AA | 10 (2) | 12 (1) |
|  | OR (95% CI)* | ref | 1.0 (0.8-1.2) |
|  | OR (95% CI)† | ref | 1.0 (0.8-1.3) |
| IS indicates ischemic stroke; SNP, single-nucleotide polymorphism; OR, odds ratio; CI, confidence interval. An additive model in binary logistic regression was used. *Adjusted for age, and sex. †Adjusted for age, sex, hypertension, diabetes mellitus, and smoking. | | | |

**Haplotype blocks in *F11***

Graphic representation of the linkage disequilibrium (LD) structure in *F11* for the analyzed tagSNPs is depicted in Fig. S1. The LD blocks were defined using Haploview 4.1 and the Solid Spine of LD algorithm.

| 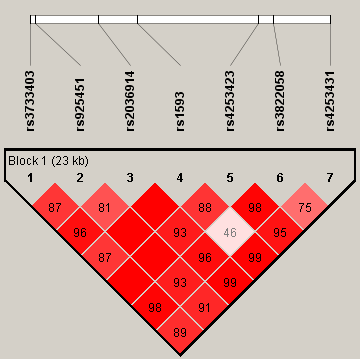 |
| --- |
| **Figure S1.** Linkage disequilibrium (LD) plot of the seven tagSNPs in *F11*. One haplotype block was determined by the Solid Spine of LD algorithm. Graphic representation of the LD structure is based on pairwise D’. |

**Genetic variation in *F11* and the etiologic subtypes of IS**

Genotype frequencies for the seven *F11* tagSNPs in the controls and in the four major etiologic subtypes of IS, and ORs and 95% CI for the subtypes in SAHLSIS are presented in Table S4.

| **Table S2.** Genotype frequencies, *n* (%), for the seven *F11* tagSNPs in controls and in the four major IS subtypes in the discovery sample SAHLSIS, as well as ORs and 95% CI for the subtypes. | | | | | | |
| --- | --- | --- | --- | --- | --- | --- |
|  | | **Control**  (n=668) | **LVD**  (n=111) | **SVD**  (n=165) | **CE stroke**  (n=151) | **Crypt**  (n=206) |
| **rs3733403** | CC | 500 (75) | 83 (76) | 134 (82) | 121 (81) | 156 (76) |
|  | CG | 150 (23) | 26 (24) | 29 (18) | 26 (17) | 46 (22) |
|  | GG | 15 (2) | 0 (0) | 1 (1) | 3 (2) | 3 (1) |
|  | OR (95% CI)* | ref | 0.9 (0.6-1.3) | 0.7 (0.5-1.0) | 0.8 (0.5-1.1) | 0.9 (0.7-1.3) |
|  | OR (95% CI)† | ref | 0.8 (0.5-1.4) | 0.6 (0.4-1.0) | 0.8 (0.5-1.2) | 0.9 (0.6-1.3) |
| **rs925451** | GG | 243 (37) | 34 (31) | 54 (33) | 48 (32) | 63 (31) |
|  | GA | 327 (49) | 55 (50) | 81 (49) | 72 (48) | 102 (50) |
|  | AA | 94 (14) | 20 (18) | 29 (18) | 30 (20) | 40 (20) |
|  | OR (95% CI)* | ref | 1.2 (0.9-1.7) | 1.2 (0.9-1.5) | 1.3 (1.0-1.6) | 1.3 (1.0-1.6) |
|  | OR (95% CI)† | ref | 1.2 (0.9-1.8) | 1.1 (0.8-1.4) | 1.3 (1.0-1.7) | 1.3 (1.0-1.6) |
| *Table S2 continued* | | | | | | |
| **rs2036914** | GG | 204 (31) | 38 (35) | 50 (31) | 52 (35) | 78 (38) |
|  | GA | 332 (50) | 52 (48) | 82 (50) | 68 (45) | 100 (49) |
|  | AA | 130 (20) | 19 (17) | 31 (19) | 30 (20) | 28 (14) |
|  | OR (95% CI)* | ref | 0.9 (0.7-1.2) | 1.0 (0.8-1.3) | 0.9 (0.7-1.2) | 0.8 (0.6-1.0) |
|  | OR (95% CI)† | ref | 0.8 (0.6-1.2) | 1.0 (0.8-1.4) | 0.9 (0.7-1.2) | 0.8 (0.6-1.0) |
| **rs1593** | AA | 499 (75) | 77 (71) | 130 (79) | 123 (82) | 172 (83) |
|  | AT | 155 (23) | 30 (28) | 34 (21) | 26 (17) | 32 (16) |
|  | TT | 12 (2) | 2 (2) | 0 (0) | 1 (1) | 2 (1) |
|  | OR (95% CI)* | ref | 1.2 (0.8-1.8) | 0.7 (0.5-1.1) | 0.7 (0.4-1.0) | 0.6 (0.4-0.9) |
|  | OR (95% CI)† | ref | 0.8 (0.5-1.4) | 0.7 (0.5-1.1) | 0.7 (0.4-1.0) | 0.6 (0.4-0.9) |
| **rs4253423** | AA | 474 (71) | 84 (79) | 113 (70) | 110 (73) | 145 (71) |
|  | AG | 173 (26) | 21 (20) | 42 (26) | 37 (25) | 55 (27) |
|  | GG | 18 (3) | 2 (2) | 6 (4) | 3 (2) | 4 (2) |
|  | OR (95% CI)* | ref | 0.7 (0.5-1.1) | 1.1 (0.8-1.5) | 0.9 (0.6-1.3) | 1.0 (0.7-1.3) |
|  | OR (95% CI)† | ref | 0.8 (0.4-1.3) | 1.1 (0.8-1.6) | 0.9 (0.6-1.2) | 1.0 (0.7-1.4) |
| **rs3822058** | GG | 302 (45) | 61 (56) | 69 (42) | 75 (50) | 98 (48) |
|  | GA | 293 (44) | 40 (37) | 79 (48) | 60 (40) | 88 (43) |
|  | AA | 71 (11) | 8 (7) | 16 (10) | 15 (10) | 19 (9) |
|  | OR (95% CI)* | ref | 0.7 (0.5-1.0) | 1.1 (0.8-1.4) | 0.9 (0.7-1.2) | 0.9 (0.7-1.2) |
|  | OR (95% CI)† | ref | 0.8 (0.6-1.2) | 1.1 (0.9-1.5) | 0.8 (0.6-1.1) | 0.9 (0.7-1.2) |
| **rs4253431** | GG | 506 (76) | 88 (81) | 120 (73) | 116 (77) | 158 (77) |
|  | GA | 150 (23) | 19 (17) | 42 (26) | 33 (22) | 43 (21) |
|  | AA | 10 (2) | 2 (2) | 2 (1) | 1 (1) | 3 (1) |
|  | OR (95% CI)* | ref | 0.8 (0.5-1.3) | 1.1 (0.8-1.6) | 0.9 (0.6-1.3) | 0.9 (0.7-1.3) |
|  | OR (95% CI)† | ref | 0.9 (0.5-1.5) | 1.2 (0.8-1.7) | 0.8 (0.6-1.3) | 1.0 (0.7-1.4) |
| IS indicates ischemic stroke; SNP, single-nucleotide polymorphism; OR, odds ratio; CI, confidence interval; LVD, large-vessel disease; SVD, small-vessel disease; CE stroke, cardioembolic stroke; Crypt, cryptogenic stroke. An additive model in binary logistic regression was used. *Adjusted for age, and sex. †Adjusted for age, sex, hypertension, diabetes mellitus, and smoking. | | | | | | |

**Genetic variation in *F11* and Kininogen 1 (*KNG1*)**

FXI circulates in the blood in complex with high-molecular-weight kininogen (encoded by *KNG1*), and variation in *KNG1* was recently shown to associate with plasma levels of FXI [[10](#_ENREF_10)]. Therefore, we checked whether any *F11* SNP is in LD with a SNP in *KNG1*. However, none of the seven investigated SNPs in the present study are in LD with any *KNG1* SNP (HapMap and 1000Genomes data).

**Supplemental References**

1. von Elm E, Altman DG, Egger M, Pocock SJ, Gotzsche PC, et al. (2008) The Strengthening the Reporting of Observational Studies in Epidemiology (STROBE) statement: guidelines for reporting observational studies. J Clin Epidemiol 61: 344-349.

2. Wilhelmsen L, Johansson S, Rosengren A, Wallin I, Dotevall A, et al. (1997) Risk factors for cardiovascular disease during the period 1985-1995 in Goteborg, Sweden. The GOT-MONICA Project. J Intern Med 242: 199-211.

3. Jood K, Ladenvall C, Rosengren A, Blomstrand C, Jern C (2005) Family history in ischemic stroke before 70 years of age: the Sahlgrenska Academy Study on Ischemic Stroke. Stroke 36: 1383-1387.

4. Adams HP, Jr., Bendixen BH, Kappelle LJ, Biller J, Love BB, et al. (1993) Classification of subtype of acute ischemic stroke. Definitions for use in a multicenter clinical trial. TOAST. Trial of Org 10172 in Acute Stroke Treatment. Stroke 24: 35-41.

5. Olsson S, Holmegaard L, Jood K, Sjögren M, Engström G, et al. Genetic Variation within the Interleukin-1 Gene Cluster and Ischemic Stroke. Accepted for publication in Stroke June 2012.

6. Redfors P, Jood K, Holmegaard L, Rosengren A, Blomstrand C, et al. (2012) Stroke subtype predicts outcome in young and middle-aged stroke sufferers. Acta Neurol Scand.

7. Hallstrom B, Jonsson AC, Nerbrand C, Petersen B, Norrving B, et al. (2007) Lund Stroke Register: hospitalization pattern and yield of different screening methods for first-ever stroke. Acta Neurol Scand 115: 49-54.

8. Li C, Engstrom G, Hedblad B, Berglund G, Janzon L (2005) Risk factors for stroke in subjects with normal blood pressure: a prospective cohort study. Stroke 36: 234-238.

9. Zia E, Hedblad B, Pessah-Rasmussen H, Berglund G, Janzon L, et al. (2007) Blood pressure in relation to the incidence of cerebral infarction and intracerebral hemorrhage. Hypertensive hemorrhage: debated nomenclature is still relevant. Stroke 38: 2681-2685.

10. Sabater-Lleal M, Martinez-Perez A, Buil A, Folkersen L, Souto JC, et al. (2012) A genome-wide association study identifies KNG1 as a genetic determinant of plasma factor XI Level and activated partial thromboplastin time. Arterioscler Thromb Vasc Biol 32: 2008-2016.
